# Routine Fluorescence Imaging to Detect Wound Bacteria Reduces Antibiotic Use and Antimicrobial Dressing Expenditure While Improving Healing Rates: Retrospective Analysis of 229 Foot Ulcers

**DOI:** 10.3390/diagnostics10110927

**Published:** 2020-11-10

**Authors:** Nadine Price

**Affiliations:** Podiatry—Gillian Hanson Diabetes Centre, Whipps Cross Hospital, Waltham Forest ICD, North East London NHS Foundation Trust, London E11 1NR, UK; nadine.price2@nhs.net

**Keywords:** dressing selection, diabetic foot ulcer, antimicrobial, fluorescence imaging, wound assessment, bacteria, MolecuLight

## Abstract

Foot ulcers and their bacterial burden produce a significant strain on the National Healthcare System (NHS). Subjectivity of wound infection assessment makes appropriate dressing selection challenging. To aid point-of-care detection of bacterial burden, a fluorescence imaging device (MolecuLight i:X) was introduced to the Whipps Cross Hospital Podiatry clinic. This retrospective pre/post-analysis evaluated how implementation of fluorescence imaging impacted (1) antimicrobial dressings and antibiotics use and (2) wound healing rates. Over a 2-year period 229 lower extremity wounds were treated. Wound-related outcomes and antimicrobial dressing costs were quantified over 1-year before (2018/2019) and after (2019/2020) incorporating fluorescence imaging into routine practice. The period of fluorescence imaging saw a 27% increase in the number of wounds seen, yet annual antimicrobial dressing expenditure decreased by 33%. Implementation of fluorescence imaging was also associated with a 49% decrease in prescription of antimicrobial dressings, a 33% decrease in antibiotic prescriptions, and a 23% increase in wound healing rates within 12-weeks (48% vs. 39%), likely due to earlier bacterial detection and improved wound hygiene. This increased healing rate is projected to decrease annual wound costs by 10% (£762 per patient). Routine bacterial imaging appears to diminish clinical and economic burden to patients and the NHS.

## 1. Introduction

Diabetic foot ulcers (DFUs) are relatively common complications of diabetes. Diabetes may result in microvascular and macrovascular damage, along with neuropathy that frequently leads to non-healing ulcers [1,2]; up to 7% of people living with diabetes in the UK currently have or have had a foot ulceration [3]. DFUs are typically chronic wounds, with some ulcers persisting for months while others never heal or lead to amputation. Five-year mortality rate of individuals with diabetic foot ulceration is as high as 40% [4]. Management and care of these complex wounds produces a significant strain on the healthcare system.

Diabetic foot ulcers represent a large and ever-increasing financial burden on the National Health Service (NHS). A recent study estimated the cost to treat diabetes-induced ulceration and amputation in England in 2014–2015 at £837–£962 m annually [5]. This is equivalent to almost £1 in every £100 spent by the NHS in England and is higher than the estimated NHS expenditure on breast, prostate, and lung cancers combined [6]. Approximately 90% of diabetic foot care costs in England are related to ulcers; average foot ulcer cost to the NHS was £7800 annually in 2018 [7]. Healing time is the largest driver of this cost [7]. With prolonged wound duration, more resources are required to manage the wound. Among them, antimicrobial dressings used to manage bacterial burden in these complex wounds account for approximately £110 million per year spent in primary care in England, with more than £20 million spent on silver dressings alone [8]. With frequent government cuts in budgets and NHS spending, clinical managers and clinicians are encouraged to seek ways to help reduce costs and control health care budgets while still providing safe and effective care for patients.

Current evidence-based practice for management of diabetic foot ulcers recommends that treatment selection be based on assessment of signs and symptoms of wound infection, along with information about the personal experiences and preferences of the patient. Infection classification systems such as those produced by the International Working Group on the Diabetic Foot (IWGDF) [9] have been developed to help characterise and guide infection management. However, these clinical signs and symptoms of infection are subjective and can go undetected and are often lacking in wounds with high bacterial loads [10], causing delays in diagnosis and appropriate treatment. As such, assessment and dressing selection remain primary clinical challenges in wound management [11,12]. Suboptimal assessment contributes to delays in wound healing and wasted resources [13]; alternative solutions that guide treatment by providing objective diagnostic information at the point-of-care are required to enhance dressing selection and improve patient outcomes.

The MolecuLight i:X fluorescence imaging device (MolecuLight Inc. Toronto, Canada) is a handheld non-contact imaging device that aids in identification of wounds with high bacterial load. The device emits a safe violet light that enables visualisation of fluorescence produced by bacteria and tissues [14]. The presence of red or cyan fluorescence provides information on clinically relevant levels of bacteria (>10^4^ CFU/g) in and around the wound; this information can be used to target cleaning and debridement [15]. The real-time information on wound bioburden provided by fluorescence imaging can address diagnostic uncertainty and ensures judicious selection of wound dressings or use of antibiotics.

The diabetic foot team at Whipps Cross University Hospital have been using fluorescence imaging in clinic for over 12 months as part of routine standard clinical care. Both clinician and patient experiences have been very positive, but we were particularly interested in evaluating how the addition of real-time fluorescence imaging to the standard wound assessment impacted: (1) use of antimicrobial dressings, as this represents our highest cost burden in relation to dressing expenditure and (2) healing rates, the highest drivers of wound costs within the NHS. In this retrospective observational pre/post-intervention analysis, we aimed to evaluate how the addition of fluorescence imaging to standard wound assessment impacted dressing selections and antibiotic prescriptions for complex foot wounds over a two-year period, and whether 12-week healing rates were impacted.

## 2. Materials and Methods

### 2.1. Standard of Care

All patients received care in line with the NICE clinical guideline NG19 [16] as a basic minimum. This included appropriate offloading, infection management, metabolic control support, wound management, and vascular assessment (doppler ultrasound). Patients also had access to the multi-disciplinary team which includes a vascular surgeon, diabetologist, podiatrist and microbiologist as and when necessary depending on their clinical need.

### 2.2. Fluorescence Imaging Procedure

**Figure 1:**
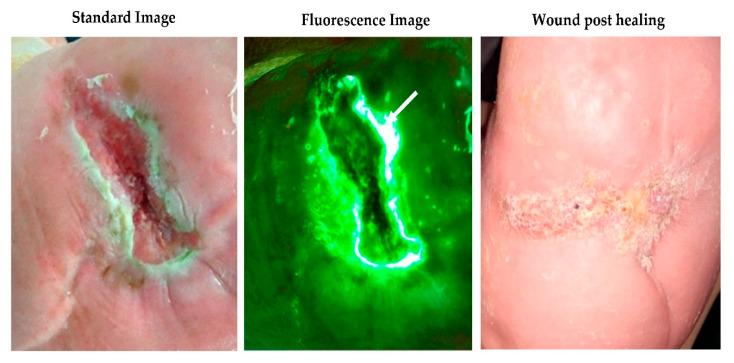
Cyan fluorescing bacterium (white arrows) indicating *Pseudomonas aeruginosa* on a diabetes foot ulcer overlying a midfoot Charcot foot deformity with subsequent standard imaging showing the wound fully healed 12 weeks later.

**Figure 2:**
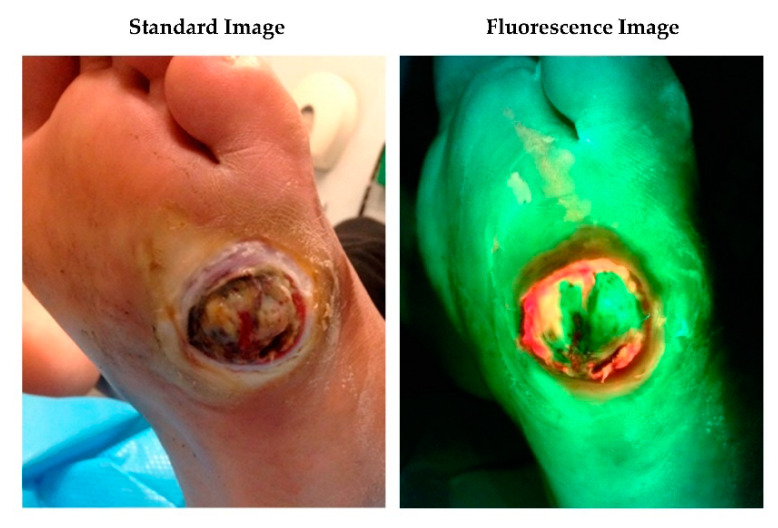
34-year-old male with extremely poorly controlled type 1 diabetes presented to the foot clinic with a 3-week history of a ‘blister’ underneath his left foot. Analysis of a deep tissue sample taken using a scalpel and forceps and targeted to the region of red fluorescence, confirmed + Group B Streptococcus, +Coagulase-negative staphylococci, +*Staphylococcus aureus*, ++*Streptococcus oralis* present in the wound. Based on the fluorescence imaging information, overall clinical presentation and x-rays confirming active osteomyelitis, he was admitted to hospital immediately and started on the appropriate intravenous antibiotics. He underwent a left 4th and 5th digital amputation and debridement approximately 5 days later but went on to make a full recovery with no further complications.

The handheld fluorescence imaging device (MolecuLight i:X, MolecuLight Inc. Toronto, Canada) visualises bacteria in the wound and peri-wound using 405nm wavelength safe violet light [14]. Fluorescence is detected from 28 common wound pathogens and is also detected from bacteria within biofilms [17]. Red fluorescence is detected from porphyrin producing-bacteria (most wound pathogens [17]) while pyoverdine-producing bacteria such as *Pseudomonas aeruginosa* are associated with a cyan fluorescent signal [18]. Red or cyan fluorescence signals are indicative of bacteria at loads >10^4^ CFU/g (moderate to heavy growth) [10,14,19]. The fluorescent signals captured by imaging are displayed on a digital touch screen and interpreted by the wound clinician without delay, to identify any regions of concern and determine an appropriate treatment plan. All acquired images and videos can then be browsed in the image library and uploaded to electronic patient records as required. Figure 1 and Figure 2 show examples of red and cyan fluorescing bacteria in diabetic foot ulceration, Figure 2 also details the microbiology results following guided tissue sampling using bacterial fluorescence imaging.

**Figure 3:**
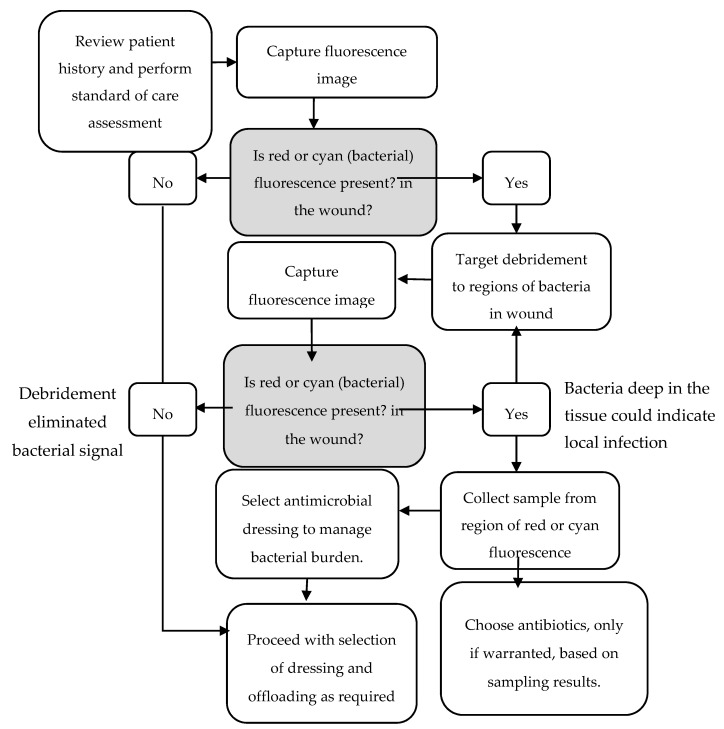
This decision tree describes how fluorescence imaging information impacted treatment selection. The presence of red or cyan fluorescence, indicative of moderate-to-heavy bacterial load, after debridement, prompted the clinician to perform additional debridement or wound sampling, as needed. Fluorescence information informed dressing selection. Antibiotics were prescribed only if indicated from sampling results except in cases where there were other clear signs or symptoms of infection present.

Real-time detection of fluorescence signals indicative of clinically significant levels of bacteria can help a clinician evaluate whether an antimicrobial dressing should be included as part of the treatment plan. Figure 3 describes a clinical decision tree that uses information provided by fluorescence imaging to inform clinical decision making. This has become the standard of care at our site since incorporating fluorescence imaging. Table 1 describes the criteria for using fluorescence imaging.

### 2.3. Extraction of Data on Antimicrobial Dressing Use and Cost Analysis

All patients who attended the foot clinic for wound care between April 2018 and March 2020 were included in the analysis. The MolecuLight i:X was introduced into the diabetic foot clinic in April 2019, therefore the 12 months prior to this are denoted as Year 1 and 12 months after this date are denoted Year 2. The data on dressing expenditure was extracted from monthly online non-prescription ordering service reports. The data regarding patient demographics, healing rates and antibiotic prescriptions were extracted from our electronic record keeping database. Although the clinic primarily provides treatment for those patients with diabetes, a small percentage of the caseload included in the data analysis were patients with lower extremity wounds originating from other causes i.e., limb ischemia. Patients who attended the clinic for other non-wound reasons (e.g., Charcot monitoring and associated treatment) were excluded. The antimicrobial dressings included in the analysis were all either silver or iodine-based dressings. There were no new antimicrobial dressings added to the formulary over the two-year period.

## 3. Results

### 3.1. Patient Demographics and Throughput

In the 12 months prior to use of the fluorescence imaging procedure for detection of bacteria, 92% of patients treated had diabetes, average age of patients was 58.4 years old and more male than female patients were treated (51/21); (Table 2). Average patient age and ratio of male to female patients remained similar between years 1 and 2, while percentage of patients with diabetes was slightly lower in year 2 compared to Year 1 (83% vs. 92%). Retrospective analysis found that 27% more wounds were treated in Year 2 compared to Year 1 (128 wounds vs. 101) and 7% more patients received treatment (77 vs. 72), despite similar clinic capacity, i.e., hours and staffing.

### 3.2. Antimicrobial and Antibiotic Usage and 12-Week Healing Rates

Baseline data from year one revealed that 39% of wounds treated in the clinic healed within 12 weeks (Table 2). Two thirds of patients were prescribed antibiotics to manage wound infection at some point during their care, and 85% of patients were prescribed antimicrobial dressings to manage infection and high bacterial burden. During this 12-month period, 18% of patients had a wound-related amputation.

In Year 2, incorporation of the fluorescence imaging procedure as part of routine wound care (Figure 3) was associated with a 33% decrease in the percentage of patients requiring antibiotic prescription compared to the previous year. This was not associated with an increased reliance on antimicrobial dressings, as the percentage of patients prescribed these dressings decreased by a notable 49%. This decrease in antimicrobial usage coincided with a 23% increase in proportion of wounds that healed within a 12-week period (48% in Year 2 vs. 39% in Year 1), and an 11% decrease in patients requiring amputation over the course of Year 2 (16% vs. 18%). This suggests that earlier bacterial detection through routine imaging combined with wound hygiene interventions was an effective diagnostic and treatment pairing in this population.

### 3.3. Antimicrobial Expenditure

The fluorescence imaging procedure was introduced into the diabetic foot clinic in April 2019. In the 12 months prior to this (2018/19) the total expenditure on antimicrobial dressings was £2291 (Table 2). A 33% reduction in antimicrobial dressing expenditure (£1531) was observed in the 12 months after the fluorescence imaging procedure was incorporated as part of standard practice when assessing wounds. This occurred despite a 7% increase in patient volume and 27% increase in the total number of wounds treated over the course of the year in which the fluorescence imaging procedure was introduced and utilised (Table 2). To account for the difference in number of wounds treated over the course of 2 years, antimicrobial spending was normalised by the number of wounds treated. Per wound, antimicrobial spending fell by 47% from £22.68 to £11.96 after introduction of point-of-care bacterial fluorescence imaging into routine practice.

### 3.4. Estimating Impact of Healing Rates on Total Wound Care Expenditure

To estimate the impact of routine fluorescence imaging on total annual wound care costs per patient, the observed healing rate data from Year 1 and Year 2 was multiplied by the known costs of wound care reported by Guest et al. [7]. Their analyses, based on a retrospective study of 130 DFU patients, reported the mean cost to the NHS of care for a foot wound over 12-months to average £7800. This cost was driven largely by the healing rate over a 12-month period; the cost of care for an unhealed foot ulcer (£8786) was found to be 4 times higher than that of a healed foot ulcer (£ 2138), while wounds resulting in amputations cost the NHS £16,941 annually on average. These varying costs were applied to the empirical healing rates observed in Year 1 and Year 2 to estimate the impact of fluorescence imaging on total NHS wound expenditure in this patient population (Table 3). This modeling incorporates the amputation rates observed in year 1 and 2 and assumes the 12-week healing rates observed in Years 1 and 2 were consistent over 12 months.

In Year 1, with 39% of wounds healed within 12-weeks, the 12-month total wound care costs were computed to average £7662, similar to the 12-month average cost of wound care reported by Guest et al. [7]. In Year 2, the 23% increase in healing rates resulted in estimated total cost of care averaging £6900 per wound, a 10% decrease in costs (savings of £762 per wound). Given that 128 wounds were treated over the course of Year 2, the total annual Year 2 savings is estimated at £97,536.

## 4. Discussion

Prescription of antimicrobial dressings in the UK has steadily increased in the last two decades [20], due in part to uncertainty relating to detection of bacterial burden in wounds. High bacterial burden is well established to delay healing [21,22]. More objective methods to detect wound bacterial burden during wound assessment may facilitate judicious use of antimicrobials and antibiotics [23]. In this study, we conducted a retrospective analysis to evaluate how routine use of fluorescence imaging, which detects elevated bacterial burden in wounds at point-of-care, impacted dressing selection and wound outcomes. To our knowledge, this is the first retrospective study reporting healing rates and direct cost savings associated with use of fluorescence imaging.

A decrease in the proportion of patients prescribed antimicrobial dressings (−49%) and antibiotics (−33%) was observed with routine use of fluorescence imaging in Year 2. The reason for this could not be conclusively determined in this retrospective analysis. However, it is speculated that this decrease, as well as the improved healing rates, is due in large part to earlier detection of wound bacteria at a stage when bacterial burden could be effectively eliminated through mechanical removal (e.g., debridement). Debridement, which effectively removes dead and bacteria-laden tissue, is standard of care and essential to wound healing; wounds debrided more frequently heal more rapidly [24], and frequent debridement is needed to disrupt bacterial biofilms [25,26]. It is not surprising, therefore, that targeting debridement to regions of red (bacterial) fluorescence is associated with faster wound area reduction rates [27]. Larger studies on healing impact of routine fluorescence imaging have been lacking, though clinical trials have reported that fluorescence images impacted the extent of debridement in greater than 50% of wounds, due to detection of bacteria that was missed by standard care [10,19]. In each of those studies, the improvements were attributed to fluorescence images alerting the wound care provider to both: (1) the presence of clinically significant levels of bacteria in wounds that fall below the infection threshold or (2) infected but asymptomatic wounds. Early detection of bacteria in wounds prior to escalation to levels of infection is essential to ensure timely healing [28]. This is especially relevant among individuals with diabetes, which make up a large portion of our patient population. Often, patients with diabetes have heavily bioburdened or infected foot ulcers [29], yet frequently fail to mount the classic signs and symptoms of infection [30]. This highlights the importance of a more sensitive [10,14] and objective method of detecting bacterial burden in wounds. In this study, given that the patient characteristics between Year 1 and Year 2 were consistent, it is unlikely that the increased healing rate and decreased need for antimicrobials were due to dramatic changes to the severity of wounds seen in the clinic in Year 2. Therefore, our analysis strongly suggests that routine use of fluorescence imaging resulted in overall improved wound management and wound outcomes.

Wound care has been flagged as a major contributor to antibiotic over prescription and resistance [23]. Antimicrobial resistance is a significant issue in the UK and worldwide that continues to have devastating consequences on human health and the economy. In response to this threat, leading healthcare committees and societies from around the world [31,32] have called for improved strategies to optimise antimicrobial use. This remains a challenge in wound care due to continued reliance on subjective and variable measures of wound infection to guide antimicrobial decision making. This, combined with an increased susceptibility of wound infection among diabetic patients [29], and clinician fear of missing the infection, has driven increased use of antimicrobial dressings [20]. To reduce unnecessary use of antimicrobials, wound care stewardship guidelines have therefore called for more objective methods to detect bacterial burden at the bedside [23]. Our analysis reveals that routine use of fluorescence imaging for detection of bacterial burden at point of care is associated with reductions in both antibiotic and antimicrobial usage, suggesting that over prescription was decreased in favor of mechanical strategies, such as debridement, to manage bacterial burden. The absence of fluorescence from bacteria in fluorescence images enhanced confidence that an antimicrobial dressing may not be needed, where previously, a clinician may have made that decision based solely on clinical assessments. The same can also be said of the opposite scenario, whereby high levels of red/cyan fluorescing bacteria detected from a wound that did not appear to be clinically infected prompted sampling to be done from the region of bacterial fluorescence and selection of an antimicrobial dressing. In many instances, topical antimicrobials were utilised instead of immediately prescribing systemic antibiotics. This reduction in antimicrobial prescription was not at the detriment of healing, indicating that the objective information provided by point-of-care fluorescence imaging does indeed contribute to improved antimicrobial decision making. 

Although there are a multitude of factors that contribute to the overall costs of treating complex foot ulcers, we chose to focus on the costs of antimicrobial dressing due to their increased use in recent years, which undoubtedly has contributed to the rise in total wound care expenditures [20]. This increase is in part driven by the considerable variation in the type and costs of antimicrobial dressings available, along with the relative lack of objective evidence to guide dressing selection. Results from our 2-year retrospective analysis indicate that implementation of fluorescence imaging of bacteria as part of routine wound screening and monitoring substantially reduced costs associated with antibiotic and antiseptic dressing usage (by 33%), while simultaneously increasing wound healing rates. The observed reduction in antimicrobial expenditure has tremendous significance for our site. However, it represents less than 1% of the potential total cost savings to the NHS [7]. Wound healing rates impact many factors that contribute to the total costs of treating foot ulcers including clinical visits for routine care, infection rates, laboratory tests, hospitalisation and amputations [2,33]. Our modeling reveals that the 23% increase in wound healing rate and 11% decrease in amputation rate observed with routine use of fluorescence imaging contributes to a 10% decrease in total costs per patient, corresponding to an annual cost savings to the clinic of £ 97,536 (based on assessment of 128 wounds per year). Wound healing times are the largest driver of costs to the NHS, therefore strategies that enhance healing rates are critical for cost containment; improving diagnosis of bacterial burden in the wound is key to these efforts, based on the findings of this study. 

In addition to these observed benefits to outcome and expenditure, it should be noted that patient education and engagement during clinical visits was enhanced with the addition of the fluorescence imaging procedure and the subsequent images collected. These images are used to demonstrate clearly the need for further diagnostic testing such as tissue sampling, imaging or blood tests, in addition to the need for antibiotics when warranted and the importance of adhering to advice given and treatment plans which are made. In our clinic, patients can see for themselves exactly where any bacteria may be at the wound site and are able to monitor, in subsequent visits, whether or not the wound is improving. Studies assessing the impact of fluorescence imaging on patient education and engagement with their care would be beneficial going forwards.

## 5. Strengths and Weaknesses

To the author’s knowledge, this retrospective analysis is the largest study of fluorescence imaging of wound bacteria performed in the UK to date, and the first to compare wound healing rates and associated costs between standard of care and fluorescence-guided care periods. There were minimal exclusion criteria for the analysis and patient populations between Year 1 and Year 2 were fairly consistent. Previous UK studies were observational in design, significantly smaller and looked primarily at burn and trauma wounds, [15,34,35]. A recent NICE med tech innovation briefing [36] highlighted a need for large, UK-based multi-centre randomised controlled trials (RCT) comparing MolecuLight i:X to standard of care within NHS settings. These trials are ongoing within the NHS (e.g., clinicaltrials.gov #NCT041875636).

There are limitations to this retrospective analysis. Most variables, other than fluorescence imaging introduction to the clinic, were consistent across the 24-month evaluation period: staff was consistent, patient demographics were similar, and there were no other new technologies introduced. However, we cannot discount the possibility that other factors may have played a role in the reduced expenditure (e.g., variability from the different patient cohort). The clinic primarily encounters foot ulcers, so it is unclear whether similar cost savings would be expected for clinics treating primarily venous leg ulcers. The single centre nature of this study is a limitation, as is the type of data available for analysis; multiple sites and larger patient numbers would be ideal. However, the year 1 data for both healing and amputation rates was strikingly consistent with reports from other NHS facilities [7], suggesting that our site is representative of the typical care provided for the treatment of foot ulcers.

## 6. Conclusions

Fluorescence imaging has proven to be a useful diagnostic procedure when examining and managing complex foot wounds at the point of care. It has undoubtedly helped with decision making, supporting discussions within the wider multi-disciplinary team. The positive impact on our dressing expenditure for antimicrobials such as silver has been very welcome in the current climate and it has supported the recommended two-week challenge for the use of silver dressings in the clinical setting. Beyond these realised cost savings, the fluorescence imaging procedure has had a noticeable impact on the quality of wound care provided to patients, without detriment to our clinical workflow. Having the ability to know at point of care whether a wound has significant levels of bacteria that may be indicative of infection enables us to offer the safest care for our patients and also provides instant clinical feedback on treatment efficacy.

## Figures and Tables

**Table 1:** Criteria for using bacterial fluorescence imaging.

✓ Suspected soft tissue infection (e.g., presence of 2 or more symptoms according to IWGDF criteria [9]) ✓ Non-healing or static wound (e.g., >4 weeks duration) ✓ Wound deterioration ✓ Sampling for microbiology ✓ Monitoring progress following application of a particular dressing or commencement of antibiotics. ✓ Monitoring progress and comparing images with those taken at a previous consultation following application of a particular antimicrobial dressing or commencement of antibiotic therapy. ✓ New ulceration

**Table 2:** Retrospective analysis of 12 months before (Year 1) and after (Year 2) implementing routine fluorescence imaging of bacteria into the podiatry clinic.

	Year 1 12 Months Prior to Fluorescence Imaging of Bacteria (2018/2019)	Year 2 12 Months Post-Fluorescence Imaging of Bacteria (2019/2020)	% Change
**Patient demographics**			
**Average patient age**	58.4	60.1	
**Male/Female**	51/21	50/27	
**% of patients with diabetes**	92%	83%	
**# of wounds seen in clinic**	101	128	+27%
**# of patients**	72	77	+7%
**Outcomes and expenditures**			
**Antimicrobial dressing expenditure**	£2,291	£1531	−33%
**Antimicrobial spending normalised to # of wounds**	£22.68	£11.96	−47%
**% of wounds healed within 12 weeks**	39%	48%	+23%
**% of patients who required antibiotics for their wound**	67%	45%	−33%
**% of patients prescribed antimicrobial dressings**	85%	44%	−49%
**% of patients requiring an amputation**	18%	16%	−11%

# refers to number of wounds.

**Table 3:** Estimated annual total cost of wound care before (Year 1) and after (Year 2) implementation of the fluorescence imaging procedure.

	Healed Foot Ulcer	Amputated Foot Ulcer	Unhealed Foot Ulcer	Total Cost of Wound Care (Annual per Patient)
Estimated cost [7]	£2138	£16,941	£8786	
Year 1 standard of care	39%	18%	43%	£7662
Year 2 standard of care + fluorescence imaging	48%	16%	36%	£6900
Estimated cost savings per patient				£762
